# Up-To-Date Review About Minipuberty and Overview on Hypothalamic-Pituitary-Gonadal Axis Activation in Fetal and Neonatal Life

**DOI:** 10.3389/fendo.2018.00410

**Published:** 2018-07-23

**Authors:** Lucia Lanciotti, Marta Cofini, Alberto Leonardi, Laura Penta, Susanna Esposito

**Affiliations:** Pediatric Clinic, Department of Surgical and Biomedical Sciences, Università degli Studi di Perugia, Perugia, Italy

**Keywords:** gonadotropin, hypothalamic-pituitary-gonadal, minipuberty, oestradiol, testosterone

## Abstract

Minipuberty consists of activation of the hypothalamic-pituitary-gonadal (HPG) axis during the neonatal period, resulting in high gonadotropin and sex steroid levels, and occurs mainly in the first 3–6 months of life in both sexes. The rise in the levels of these hormones allows for the maturation of the sexual organs. In boys, the peak testosterone level is associated with penile and testicular growth and the proliferation of gonadic cells. In girls, the oestradiol levels stimulate breast tissue, but exhibit considerable fluctuations that probably reflect the cycles of maturation and atrophy of the ovarian follicles. Minipuberty allows for the development of the genital organs and creates the basis for future fertility, but further studies are necessary to understand its exact role, especially in girls. Nevertheless, no scientific study has yet elucidated how the HPG axis turns itself off and remains dormant until puberty. Additional future studies may identify clinical implications of minipuberty in selected cohorts of patients, such as premature and small for gestational age infants. Finally, minipuberty provides a fundamental 6-month window of the possibility of making early diagnoses in patients with suspected sexual reproductive disorders to enable the prompt initiation of treatment rather than delaying treatment until pubertal failure.

## Introduction

Puberty is the period of life in which a child develops secondary sexual characteristics and reproductive function. Puberty requires activation of the hypothalamic-pituitary-gonadal (HPG) axis, resulting in secretion of hypothalamic gonadotropin-releasing hormone (GnRH), which in turn stimulates secretion of luteinizing hormone (LH) and follicle stimulating hormone (FSH) by the pituitary gland and the consequent maturation of gametogenesis as well as secretion of gonadal hormones. Before the onset of puberty, the HPG axis is temporary activated in two other periods of life, i.e., in the midgestational fetus and in the newborn. In recent years, many studies in the literature have referred to this latter period as minipuberty.

Minipuberty was first described in the 1970s (1, 2), but its role is still not well understood. The aim of this review is to analyse the impact and the clinical role of minipuberty. PubMed was used to search for all relevant studies published over the last 25 years using the key words “minipuberty,” “mini-puberty,” “HPG axis,” “gonadotropins,” and “sexual hormones,” combined with “fetal life,” “newborn,” “preterm,” “small for gestational age,” “growth,” “congenital hypogonadotropic hypogonadism,” “Turner syndrome,” “Klinefelter syndrome” and “CAIS”. Additional sources were found from the references of the publications that were obtained from the search. The data obtained from studies published between 1973 and 2017 are included in this review, and the most recent research is dated March 2017.

## Hypothalamic-pituitary-gonadal (HPG) axis activation in fetal life

During embryogenesis, neurons that produce GnRH develop from the epithelium of the medial olfactory pit and move to the fetal hypothalamus by migrating along nerve fibers (3). This process occurs at ~40 days of gestation (4). Simultaneously, the pituitary gland develops and begins synthesizing both LH and FSH at 9 weeks of gestation (WG) (5), although the hormones appear in the fetal blood by 12–14 WG (6). Kisspeptin and KISS1R are factors that are involved in the regulation of fetal GnRH neuron activity. However, serum LH and FSH levels are independent of GnRH and kisspeptin at midgestation, but they become GnRH-induced after 30–31 WG (7).

The gonadotropin levels peak at midgestation in both the pituitary gland and the serum and subsequently decrease toward birth and are suppressed at term (8, 9). This pattern is probably caused by the gradual increase in the production of placental estrogens toward the end of gestation (10) that suppresses the activity of the fetal HPG axis.

Additionally, female fetuses produce higher LH and FSH levels than male fetuses (6, 11). Indeed, Debieve et al. (12) measured LH and FSH at midpregnancy (the group had median ages of 23.8 WG for the females and 22.6 WG for the males) and at term (median ages: 39.2 WG for the females and 38.9 WG for the males). Both gonadotropins were present in the first group and exhibited a clear difference between the females and males; the girls exhibited much higher levels (33.0 ± 23.2 vs. 4.4 ± 3.3 mIU/mL for LH and 54.4 ± 27.7 vs. 0.77 ± 0.49 mIU/mL for FSH). In contrast, in the term female fetuses, both LH and FSH were undetectable, and only very low FSH levels were observed in the term male fetuses. The midpregnancy gonadotropin peak coincides with the first ovarian follicle or seminiferous tubule maturation. The difference between genders is probably caused by the negative feedback that results from the higher concentrations of fetal testicular hormones (6, 13, 14). Another marked difference between the sexes is that the LH levels overcome the FSH levels in male fetuses (15), whereas the opposite situation occurs in females.

During fetal life, the masculinization of genitalia depends on the production of testosterone (T) by the Leydig cells of the fetal testicles and on its action on target organs. During the first trimester of gestation, placental human chorio-gonadotropin (hCG) induces the differentiation of testicular mesenchymal cells into Leydig cells and stimulates T production through the activation of the LH/CG receptors expressed on their surfaces (16). Indeed, mutations of the LH/CG receptor can cause the absence of virilization and feminization of the external genitalia (17). Thus, the fetal testicular T is secreted first under the control of placental hCG, and only after the 9th WG, T secretion comes under the control of pituitary LH. A clear increase in T concentration occurs between 8 and 11 WG and reaches a maximum between 11 and 14 WG. The peak level (40–580 ng/dL) is similar to the adult value (14), whereas T levels in the fetal testes can reach ~1.9–2.1 ng/mg of tissue (16). After the 20th WG, T decreases toward term (8, 14). The fetal testicles also express FSH receptors that probably control Sertoli cell proliferation, although only few studies exist regarding the effects of FSH (18–20). Anti-mullerian hormone (AMH) that is produced by the Sertoli cells in the fetal testes causes the regression of the mullerian ducts, which prevents the formation of internal feminine genitalia. T favors the development of male urogenital structures from the wolffian duct, such as the vas deferens, epididymis, and seminal vesicles, while the formation of the prostate, penis and scrotum is due to the active metabolite of T (dihydrotestosterone).

In female fetuses, the lack of AMH allows the mullerian ducts to develop into the fallopian tubes, uterus and upper part of the vagina (21). The development of the primordial follicle in the fetal ovaries begins before 13 WG, but the follicles are more rapidly created after 14–15 WG. The pool of primordial follicles is ~100,000 at 15 WG and then rapidly increases to reach a higher number at 34 WG (680,000). Subsequently, the pool remains stable, at least until 8 months after birth (22). This pool, which represents the foundation of female fertility, is formed when estrogen levels are high in the fetal circulation. However, the majority of estrogen production during fetal life is due to the placenta, and ovarian production can be considered irrelevant. Additionally, the roles of FSH and LH in ovarian development during pregnancy are not completely understood. It seems that normal development occurs until the 34th WG even in anencephalic fetuses. In contrast, during the last part of gestation, a marked difference can be found; in anencephalic fetuses small and growing antral follicles cannot be observed as they can in normal fetuses (23), which suggests that hypothalamic stimulation is necessary to ensure physiological ovarian development after the 7th month of gestation.

## Minipuberty and its implications in healthy infants

At delivery, in healthy infants the LH and FSH levels are low in the cord blood in both sexes (24) due to the inhibitory effect of the high levels of placental estrogens. In boys, the LH level increases by ~10-fold in the few minutes after delivery, and this increase is followed by a concomitant rise in the T levels that lasts for ~12 h (25, 26). In girls, this increase does not occur.

**Figure 1:**
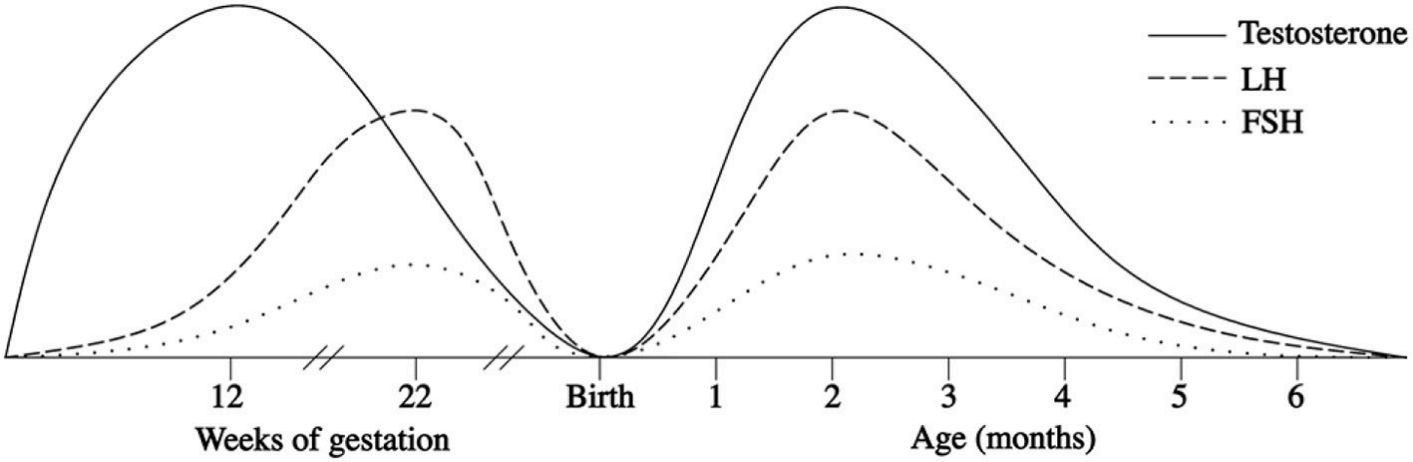
Patterns of fetal and postnatal luteinizing hormone (LH), follicle stimulating hormone (FSH) and testosterone (T) secretion in males.

**Figure 2:**
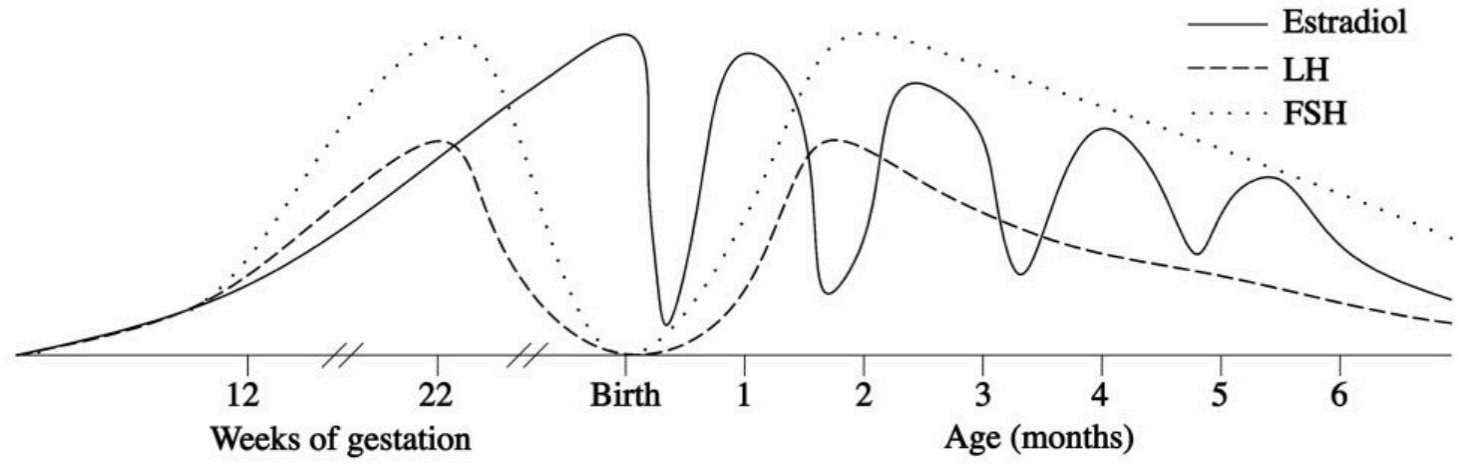
Patterns of fetal and postnatal luteinizing hormone (LH), follicle stimulating hormone (FSH) and oestradiol secretion in females.

In the first few days after birth, the fall in circulating placentally produced steroids causes a progressive lack of negative feedback on the neonatal GnRH pulse generator. In this manner, the activity of the HPG axis is reinitiated, and gonadotropin levels begin rising between days 6 and 10 after birth (27, 28). In infant boys, the serum LH level reaches the pubertal range by 1 week of age, the peak is detected between the 2nd and 10th weeks of life, and the level then decreases to the prepubertal range by 4–6 months (27–30) (Figure 1). Female infants have lower peak LH values, but the pattern is similar (Figure 2) (27–29). In contrast, the FSH levels are higher in females than in males and peaking between 1 week and 3 months. Subsequently, in males, the FSH values gradually decrease to the prepubertal range within 4 months of age, whereas in females, these values remain elevated until to 3–4 years of age (27, 29, 31).

Table 1 summarizes sex differences and the patterns of basal LH/FSH in the first month of life. In male neonates, the pattern of T secretion is similar to that of LH secretion: T is low in the cord blood, gradually increases to peak at 1–3 months and then declines to prepubertal values by 6–9 months of age (2, 27, 28, 30, 32). Similarly, the number of Leydig cells in the testicular tissue increases considerably until the third month and then gradually decreases due to apoptosis of the fetal Leydig cells (33–36). Sertoli cells also develop in the first months after birth under the stimulation of FSH (37), but they do not express androgen receptors during infancy, and therefore spermatogenesis does not occur (38). These cells secrete AMH in male neonates: the highest levels are observed at 3 months, and the levels subsequently decline and remain at relatively stable levels throughout childhood until puberty at which point AMH progressively decline to the adult level of 3–4% of the infant level (39). The absence of androgen receptors explains why AMH levels remain elevated in the presence of the high T levels during early infancy (38, 40). Additionally, in prepubertal boys, Sertoli cells secrete inhibin B, which represents a marker of Sertoli cell function. This hormone, which is already present in the cord blood, increases soon after birth, peaks at 3–6 months of age, reaches greater values then those observed in adults (mean +/– SD: 378 +/– 23 pg/mL), and remains elevated until at least the age of 15 months (28, 32, 41).

**Table 1:** Luteinizing hormone (LH) and follicle stimulating hormone (FSH) values (means ± SD) in neonates.

**Age (days)**	**LH in males**	**LH in females**	**FSH in males**	**FSH in females**
1–5	0.39 ± 0.48	0.48 ± 0.66	0.96 ± 0.60	2.00 ± 1.37
6–10	2.31 ± 2.29	0.45 ± 0.33	2.91 ± 4.38	2.44 ± 2.52
11–15	3.55 ± 2.84	1.58 ± 1.28	3.71 ± 2.69	8.16 ± 4.27
16–20	4.13 ± 2.76	1.03 ± 1.39	2.63 ± 1.45	1.62 ± 1.05
21–25	2.86 ± 1.51	0.46 ± 0.25	2.50 ± 1.51	7.07 ± 5.92
26–28	2.22 ± 2.37	2.75 ± 2.39	2.25 ± 0.81	9.74 ± 9.89

*Values of LH and FSH are in mIU/mL*.

*Adapted from Schmidt and Schwarz HP (27)*.

Minipuberty has been associated with physiological gonadal development processes, such as penile and testicular growth and the proliferation of gonadic cells. Cortes et al. (42) found that penile length is positively correlated with serum T and increases from birth (mean +/– SD, 3.49 +/– 0.4 cm) to 3 years of age with the highest growth velocity occurring from birth to 3 months (1 mm/month). In contrast, testicular volume increases significantly in the first 5–6 months of life, from 0.27 to 0.44 cm^3^, and the volume subsequently decreases to 0.31 cm^3^ at ~9 months (43). This pattern is positively correlated with FSH levels (30), which is probably due to the proliferation of Sertoli cells in the seminiferous tubules (37). Additionally, Hadziselimovic et al. proposed that germ cells can differentiate into adult (Ad) spermatogonia due to the transient activation of the HPG axis during the first months of life (44).

Thus, the first months of life are fundamental for the development of the male reproductive organs. In contrast, it is not completely clear whether these months are also important for the reproductive functions of girls. At birth, oestradiol levels are high in the cord blood of both sexes. Umbilical cord estrogen concentrations depend on gestational age, the mode of delivery, pregnancy complications, and twinning, but not on infant sex (45). In a study conducted by Troisi et al. (46), the mean oestradiol values measured in cord blood were found to be 11,941 pg/ml in females and 12,782 pg/ml in males, and the difference between gender was not significant. During the first postnatal days, oestradiol levels gradually decrease, but after 1 week of age, they increase in girls only and remain high in the subsequent period (47–49) until at least the 6th month of life. Similar observations were made by Bidlingmaier et al. (50), who found the higher oestradiol concentration in the ovaries of 1- to 6-month-old girls compared with those at the end of the first year in postmortem samples. At 3 months of age, the median serum oestradiol level in girls is 30.0 pmol/L (range < 18–100) (47). However, individual oestradiol levels in girls exhibit considerable fluctuation in the first months of life, which may reflect the cycles of maturation and atrophy of the ovarian follicles. Indeed Kuiri-Hanninen et al. (31) reported increased numbers of antral follicles on ovarian ultrasonography in infant girls, and this fact corresponds to parallel elevations of oestradiol (48, 49) and AMH levels (31, 51). Indeed, in infant girls, there is a marked rise of AMH levels at 3 months of age (15 pmol/L; 4.5–29.5 pmol/L) compared with the levels found in cord blood (2 pmol/L; 2–15.5 pmol/L) and at 1 year of age (51). This fact may demonstrate the postnatal proliferation of granulosa cells, which produce AMH, and the contemporary development of the ovarian follicles, which probably occurs in response to the parallel FSH surge (31, 51).

The mammary glands and the uterus are also certainly estrogen target tissues in the fetus and in newborn. At birth, most full term babies of both sexes have palpable breast tissue (52) that probably results from in-uterus stimulation from placental estrogens. However, in the following months, the breast tissue in females remains larger and persists longer (52). In boys, the mammary gland diameter gradually decreases until the 6th month, whereas in full-term girls, it remains large, reflecting the activity of endogenous estrogens (48). In contrast, the uterine length increases primarily during pregnancy due to the hormones that cross the placental barrier, and after birth, it is longest at day 7 in full-term babies and then steadily decreases toward the third month and remains fairly unchanged until the second year (48). Therefore, the role of minipuberty in girls is still controversial and partially unknown.

## Minipuberty in premature infants and in those small for gestational age (SGA)

Postnatal HPG axis activation also occurs in premature infants and is even stronger and more prolonged in time than in full-term infants (53, 54). Kuiri-Hanninen et al. (30) recently compared full-term (FT) and preterm (PT) males by measuring urinary gonadotropins and T in serial urine samples and comparing the results with testicular and penile growth. The trends of LH and T secretion are similar among the groups, but the levels are significantly higher in PT than in FT males when measured at 7 and 30 days of age. These levels then decline in both groups, but a significant difference can still be observed at month 6. Additionally, a positive association between the level of HPG axis activation and the grade of prematurity has been found, and PT males have been associated with faster penile and testicular growth after birth, which suggests that HPG axis activation plays a role in completing genital development.

In PT females, the FSH and LH values are higher than those in FT girls (55) and exhibit a more elevated and more prolonged peak; these patterns might reflect the expression of the immaturity of a negative feedback system in the HPG axis of PT girls. The greater levels of FSH in PT females are probably due to a delay in ovarian folliculogenesis; the ovaries are still immature and do not seem to be able to produce sufficient estrogens that may inhibit gonadotropin secretion. Additionally, the FSH peak is followed by transient ovarian stimulation (which reaches a maximum at ~4 weeks of age) that results in the presence of antral follicles on ultrasonography and increases in the levels of granulosa cell–derived AMH and oestradiol (31), which are higher in the serum of PT than FT girls (49). Estrogen receptor alpha is expressed in the fetal mammary glands from ~30 weeks of gestation (56), which might explain the absence of breast development in PT infants. However, in these girls, there is a stronger association between the postnatal oestradiol surge and the growth of the mammary gland diameter and uterine length (48).

A possible clinical consequence of this intensive stimulation on the genital organs that occurs in premature infants is ovarian hyperstimulation syndrome. First described in 1985 by Sedin et al. (57) in 4 very preterm neonates, ovarian hyperstimulation syndrome is characterized by oedema of the vulva, solitary or multiple cysts in the ovaries on ultrasonography, breast growth, occasional vaginal bleeding and high serum gonadotropin and oestradiol levels. This syndrome is probably the extreme consequence of the immaturity of the negative feedback mechanisms that act on the HPG axis, and this absence of feedback results in hyperstimulation of the target organs. Several cases have been reported in the literature (58–62), and all cases indicate that it is a self-limiting disease that does not require treatment if there are no complications, but follow-up until clinical resolution is necessary.

Infants born small for gestational age (SGA) are at risk of developing metabolic and endocrinological disorders (63). It is well known that SGA children are at greater risk of type 2 diabetes and cardiovascular diseases, expecially those with high catch up growth. In fact, the fetus in nutritional deficiency constantly replans his metabolism to slow growth with relative resistance to insulin, IGF-1 and GH, that persists in childhood and adult life too (64). Besides, lower insulin sensitivity has been also associated to an increased incidence of adrenal and ovarian hyperandrogenism, clinically evident as precocious pubarche and reduced ovulation rate (65).

In females, being SGA has been associated with reduced uterine and ovary size (66), whereas, in males, SGA has been linked to infertility and reduced testicular volume and T concentrations in adult life (67). Minipuberty in SGA infants is still not well defined, and the data reported in the literature are controversial. Recently, Nagai et al. (68) conducted a longitudinal study and reported lower FSH and T, as well as higher LH concentrations, in SGA infants compared with appropriate for gestational age (AGA) infants. In contrast, in previous studies, elevated serum FSH concentrations have been detected in SGA infant girls and boys (69); similarly, higher T levels have been found in SGA than in AGA boys (70). Further studies are necessary to definitely clarify the patterns of minipuberty in SGA infants and their clinical implications.

## Minipuberty and growth

Growth is influenced by different hormones depending on the period of life. In fact, in the first years, thyroid hormones play the main role, together with insulin and glucocorticoids. By contrast, GH becomes predominant during infancy until the period of puberty, when the rise of levels of sexual hormones results in the growth spurt, which is essential for final growth and bone maturation.

Recently, new studies have described an association between minipuberty and growth, particularly in males. Indeed, sex steroids and gonadotropic hormones in the first 5 months of life seem to influence somatic development in boys during the following 6 years. Becker et al. (71) conducted a prospective study of 35 healthy infants (17 males) and reported that the surge in T during the first months of life has an influence on human somatic and adipose tissue development in childhood. Indeed, boys exhibit faster increases in weight and BMI at the age of 8 weeks than do girls. At this age, the median T level has been found to be 7.37 nmol/L, which corresponds to pubertal male values. Subsequently, other trials have been conducted with greater numbers of participants. Kiviranta et al. (72) studied 84 healthy neonates (45 of which were males). The linear growth velocity was significantly faster from birth to 6 months of age in boys than in girls, and the greatest growth velocity difference, i.e., 4.1 cm per year, was observed at 1 month of age, which is simultaneous with the peak of the postnatal gonadal activation, especially in terms of T level.

## Minipuberty and hypogonadism

In the last 20 years, studies have been conducted to establish an association between minipuberty and hypogonadotropic hypogonadism (HH), especially in males, and have found that the disease is characterized by the absence of the postnatal FSH, LH, and T surge. For this reason, minipuberty provides a short-time window of opportunity to make an early diagnosis (41). At birth, HH can be revealed by a micropenis with or without associated cryptorchidism, and male neonates exhibiting these “red flags” should undergo a single serum sample examination to identify congenital gonadotropin deficiency (73, 74). A novel study conduced in a large cohort of HH patients, in fact, confirmed the importance of identifying male genital tract anomalies in prepubertal age, in particular showing that micropenis or cryptorchidism are significantly more represented in HH resulting from Kallman syndrome (75). The best period for the measurement of the serum concentrations of reproductive hormone is between 4 and 8 weeks of life, but it can be practically performed until 6 months of age (73). Besides, a recent study suggested that testicular position increases from birth to 3 months of age and decreases thereafter, overlapping with the period of minipuberty. Therefore, testicular distance to pubic bone may be a useful biomarker of postnatal testicular function, both for Leyding and Sertoli cell activity (76).

Hadziselimovic et al. identified impaired minipuberty as the main reason for cryptorchidism-induced infertility (77). Indeed, the lack of secretion of LH and T in the perinatal period prevents the differentiation of germ cells, which results in infertility after puberty (78).

Furthermore, the importance of minipuberty has been highlighted by evidence that orchiopexy alone does not necessary improve fertility in cryptorchid males, whereas in 6-month-olds, long targeted therapy with LH-Rh analogs following successful surgery results in the normalization of the sperm parameters in adult life (79). Table 2 presents case reports and descriptions of small trials of patients with HH who were treated during the first year of life with recombinant human LH and FSH or with T in attempts to imitate physiological minipuberty. In all cases, the effects were beneficial. The substitutive therapy with T resulted in a marked increase in T level and penile length, whereas recombinant gonadotropin administration caused increase not only in T level, but also in LH, FSH, as well as inhibin B and AMH. This hormonal pattern results both in the increase of penile length and testicular volume and in fertility potential later in life. The administration of gonadotropins is safe, well tolerated and effective. Finally, new evidences suggest also the possibility of treatment with a gonadotropin-releasing hormone agonist, which induces gonocytes to differentiate into Ad spermatogonia and rescues fertility (84). As regards hypergonadotropic hypogonadism, one of the most frequent causes is Turner syndrome (TS). In these patients, perinatal FSH secretion is similar to that in healthy girls (85), however, during infancy, the pattern of FSH secretion is strictly related to karyotype. Young girls with monosomy TS exhibit a persistent elevation of FSH up to 6 years, whereas those with 45,X/46,XX mosaicism have only minimally elevated FSH values, which suggests the presence of feedback effects on the HPG axis due to retained ovarian function (86, 87).

**Table 2:** Replacement therapy for hypogonadotropic hypogonadism in the first year of life.

**References**	**Year**	**N. cases**	**Hormonal therapy**	**Clinical and hormonal outcome**
Main et al. (80)	2000	3	T	↑ T levels and penis length
Main et al. (81)	2002	1	rLH and rFSH	↑ inhibin B levels; ↑ testicular volume and penis length
Bougnères et al. (82)	2008	2	rLH and rFSH	↑ T, inhibin B and AMH levels; ↑ testicular volume and penis length
Stoupa et al. (83)	2017	6	rLH and rFSH	↑ T, inhibin B and AMH levels; ↑ penis length

*AMH, anti-mullerian hormone; rFSH, recombinant stimulating hormone; rLH, recombinant luteinizing hormone; T, testosterone*.

Contrasting data can be found in the literature about males with Klinefelter syndrome (KS). Lahlou et al. (88) found that, in their cohort of KS patients 0–3 years old, the FSH, LH, and inhibin B levels were similar to those in healthy controls with the exception of T, which exhibited a physiological increase during the first trimester, but always remained at a lower level thereafter when compared with controls. Similarly, Ross et al. underlined that the neonatal surge in T is attenuated in the KS population (89). In contrast, Aksglaede et al. (90) found elevated LH levels and high-normal serum T levels in KS infants, as well as evidence of subtle Sertoli cell dysfunction with low-normal inhibin B levels. This situation may predict the postpubertal resistance of Sertoli cells to FSH action in subjects with KS in adult life (91). Table 3 summarizes studies on minipuberty in KS.

**Table 3:** Minipuberty in Klinefelter syndrome (KS).

**References**	**Year**	**N. cases**	**N. controls**	**Age of population**	**Hormonal findings**
Lahlou et al. (88)	2004	18	215	0–3 years	FSH, LH, inhibin B, and AMH not different between groups; T ↓ in KS
Ross et al. (89)	2005	22	–	1–23 months	T ↓ in KS
Aksglaede et al. (90)	2007	10	613	3 months	↑ T, LH and FSH; inhibin B not different between groups
Cabrol et al. (91)	2011	68	215	2–750 days	T, LH, inhibin B and AMH not different between groups; normal or ↑ FSH levels in KS

*AMH, anti-mullerian hormone; LH, uteinizing hormone; rFSH, recombinant stimulating hormone; T, testosterone*.

Finally, androgen receptors also play an important role in HPG axis activity. Indeed, infants with complete androgen insensitivity who present with a mutation in the androgen receptor do not exhibit the physiological peaks in LH and consequently T during minipuberty, whereas neonates with partial androgen insensitivity exhibit high-normal levels of postnatal T and LH (92).

## Conclusions

The HPG axis is physiologically activated in the fetus during midgestation and gradually turns off toward term due to the negative feedback of placental hormones on the fetal hypothalamus. At birth, when the restriction is removed, the HPG axis reactivates, which results in a T peak in males between months 1 and 3; by contrast, the oestradiol levels in females fluctuate until 6 months of age. Minipuberty allows for the development of the genital organs and creates the basis for future fertility, but further studies are necessary to understand its exact role, especially in girls. Nevertheless, no scientific study has yet elucidated how the HPG axis turns itself off and remains dormant until puberty. Additional future studies may identify clinical implications of minipuberty in selected cohorts of patients, such as premature and small for gestational age infants. Finally, minipuberty provides a fundamental 6-month window of the possibility of making early diagnoses in patients with suspected sexual reproductive disorders to enable the prompt initiation of treatment rather than delaying treatment until pubertal failure.

## Author contributions

LL drafted the manuscript. MC and AL performed the literature review. LP supervised the project. SE revised the manuscript and made substantial scientific contributions.

### Conflict of interest statement

The authors declare that the research was conducted in the absence of any commercial or financial relationships that could be construed as a potential conflict of interest.
